# Hexamethyl 13,14-dioxapenta­cyclo­[8.2.1.1^4,7^.0^2,9^.0^3,8^]tetra­deca-5,11-diene-1,4,5,6,11,12-hexa­carboxyl­ate

**DOI:** 10.1107/S1600536812039232

**Published:** 2012-09-22

**Authors:** Alan J. Lough, Kelsey Jack, William Tam

**Affiliations:** a Department of Chemistry, University of Toronto, Toronto, Ontario, Canada M5S 3H6; b Department of Chemistry, University of Guelph, Guelph, Ontario, Canada N1G 2W1

## Abstract

In the title compound, C_24_H_24_O_14_, the stereochemistry at the cyclo­butane ring is *cis-anti-cis* and the –COOMe groups in the bicyclic rings are *syn* to each other. The mol­ecule lies on a twofold rotation axis. In the crystal, weak C—H⋯O hydrogen bonds connect mol­ecules into chains along [001], forming *R*_2_^2^(10) rings.

## Related literature

For related structures, see: Lough *et al.* (2012*a* ▶,*b* ▶). For the synthetic background, see: Ballantine *et al.* (2009 ▶). For hydrogen-bond motifs, see: Bernstein *et al.* (1995 ▶).
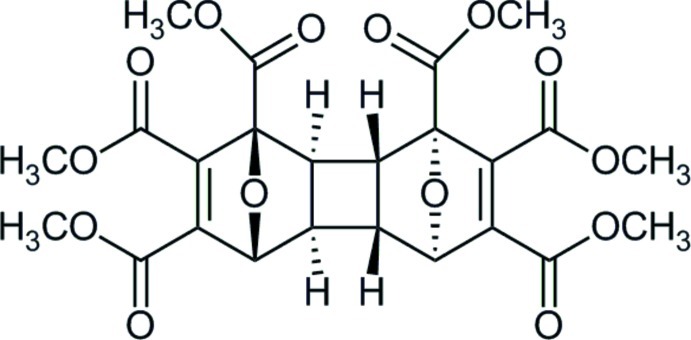


## Experimental

### 

#### Crystal data

C_24_H_24_O_14_
*M* *_r_* = 536.43
Monoclinic, 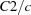

*a* = 25.1309 (18) Å
*b* = 10.0840 (7) Å
*c* = 9.5922 (7) Å
β = 95.430 (2)°
*V* = 2419.9 (3) Å^3^
*Z* = 4
Mo *K*α radiation
μ = 0.12 mm^−1^
*T* = 147 K
0.15 × 0.10 × 0.07 mm

#### Data collection

Bruker Kappa APEX DUO CCD diffractometer
Absorption correction: multi-scan (*SADABS*; Bruker, 2007 ▶) *T*_min_ = 0.711, *T*_max_ = 0.746
11038 measured reflections
2787 independent reflections
2366 reflections with *I* > 2σ(*I*)
*R*_int_ = 0.028

#### Refinement

*R*[*F* ^2^ > 2σ(*F* ^2^)] = 0.035
*wR*(*F* ^2^) = 0.098
*S* = 1.04
2787 reflections
175 parameters
H-atom parameters constrained
Δρ_max_ = 0.42 e Å^−3^
Δρ_min_ = −0.25 e Å^−3^

### 

Data collection: *APEX2* (Bruker, 2007 ▶); cell refinement: *SAINT* (Bruker, 2007 ▶); data reduction: *SAINT* (Bruker, 2007 ▶); program(s) used to solve structure: *SHELXS97* (Sheldrick, 2008 ▶); program(s) used to refine structure: *SHELXL97* (Sheldrick, 2008 ▶); molecular graphics: *PLATON* (Spek, 2009 ▶); software used to prepare material for publication: *SHELXTL* (Sheldrick, 2008 ▶).

## Supplementary Material

Crystal structure: contains datablock(s) global, I. DOI: 10.1107/S1600536812039232/hb6954sup1.cif

Structure factors: contains datablock(s) I. DOI: 10.1107/S1600536812039232/hb6954Isup2.hkl

Supplementary material file. DOI: 10.1107/S1600536812039232/hb6954Isup3.cml

Additional supplementary materials:  crystallographic information; 3D view; checkCIF report

## Figures and Tables

**Table 1:** Hydrogen-bond geometry (Å, °)

*D*—H⋯*A*	*D*—H	H⋯*A*	*D*⋯*A*	*D*—H⋯*A*
C6—H6⋯O1^i^	1.00	2.38	3.2105 (13)	140

Symmetry code: (i) 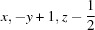.

**Fig. 1.**
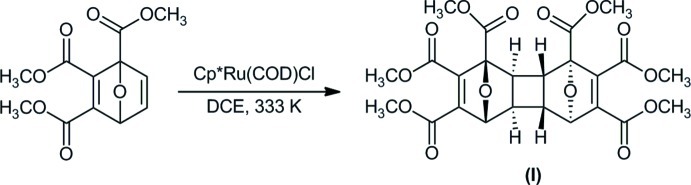
Reaction scheme

**Fig. 2.**
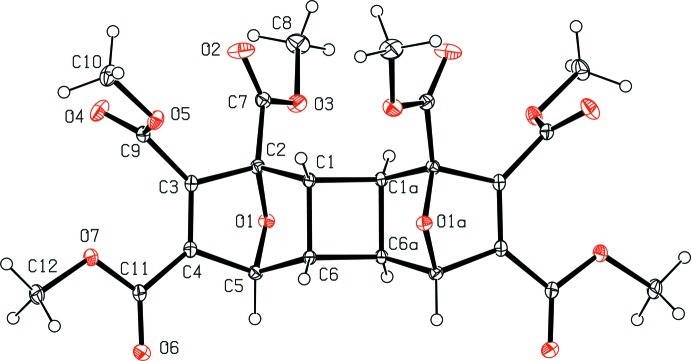
The molecular structure of (I) showing 30% probability ellipsoids (symmetry code (a): -x + 2, y, -z + 1/2).

**Fig. 3.**
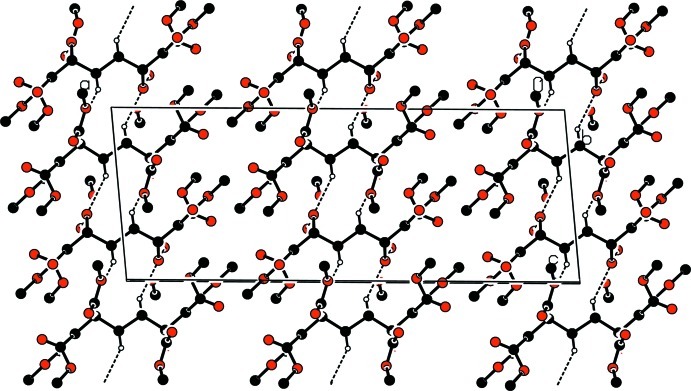
Part of the crystal structure showing weak hydrogen bonds as dashed lines.

## supplementary crystallographic information

### Comment

We have recently investigated the Ru-catalyzed isomerization and dimerization reaction of oxanorbornadiene compounds (Ballantine *et al.*, 2009). When dissolved in 1,2-dichloroethane in the presence of Cp*Ru(COD)Cl, 1,2,3-trimethyl-7-oxabicyclo[2,2,1]hepta-2,5-diene-1,2,3-tricarboxylate will dimerize into (I) (Fig. 1). The desired product was resolved using fractional crystallization in methanol. The stereochemistry and regioschemistry of the product was determined by this single-crystal X-ray analysis. The only dimer product obtained was found to have a *cis*-anti-*cis* stereochemistry at the cyclobutane ring of the dimer, and the two COOMe groups in the bicyclic rings are *syn* to each other.

The molecular structure of (I) is shown in Fig. 2. The molecule lies on a twofold rotation axis. In the crystal, weak C—H···O hydrogen bonds connect molecules into one-dimensional chains (Fig. 3) along [001] forming *R*^2^_2_(10) rings (Bernstein *et al.*, 1995).

For related structures, see the two preceding papers (Lough *et al.*, 2012*a*,*b*).

### Experimental

1,2,3-Trimethyl-7-oxabicyclo[2,2,1]hepta-2,5-diene-1,2,3-tricarboxylate (316 mg, 1.2 mmol) was weighed into an oven-dried vial, purged with nitrogen and transferred into a Dry Box. In the Dry Box, Cp*Ru(COD)Cl (10 mol%) was added to another oven dried vial and dissolved in 1,2-dichloroethane (2 ml). The Ru-catalyst was then transferred into the vial containing the 7-oxanorbornadiene. The vial was sealed with a screw cap and removed from the Dry Box. The reaction was heated at 333 K with stirring for 18 h. The crude product was purified by column chromatography (EtOAc:hexanes=2:3) followed by recrystallization in methanol to give the dimer (I). Colourless needles were grown by slow evaportation of a solution of (I) in methanol.

### Refinement

Hydrogen atoms were placed in calculated positions with C—H distances of 0.98 and 1.00 Å. They were included in the refinement in a riding-model approximation with *U*_iso_(H) = 1.2*U*_eq_(C) or 1.5*U*_eq_(C_methyl_).

### Crystal data

**Table:**

C_24_H_24_O_14_	*F*(000) = 1120
*M**_r_* = 536.43	*D*_x_ = 1.472 Mg m^−^^3^
Monoclinic, *C*2/*c*	Mo *K*α radiation, λ = 0.71073 Å
Hall symbol: -C 2yc	Cell parameters from 6078 reflections
*a* = 25.1309 (18) Å	θ = 3.0–27.5°
*b* = 10.0840 (7) Å	µ = 0.12 mm^−^^1^
*c* = 9.5922 (7) Å	*T* = 147 K
β = 95.430 (2)°	Needle, colourless
*V* = 2419.9 (3) Å^3^	0.15 × 0.10 × 0.07 mm
*Z* = 4	

### Data collection

**Table:**

Bruker Kappa APEX DUO CCD diffractometer	2787 independent reflections
Radiation source: fine-focus sealed tube	2366 reflections with *I* > 2σ(*I*)
Bruker Triumph monochromator	*R*_int_ = 0.028
φ and ω scans	θ_max_ = 27.5°, θ_min_ = 1.6°
Absorption correction: multi-scan (*SADABS*; Bruker, 2007)	*h* = −32→32
*T*_min_ = 0.711, *T*_max_ = 0.746	*k* = −13→12
11038 measured reflections	*l* = −11→12

### Refinement

**Table:**

Refinement on *F*^2^	Primary atom site location: structure-invariant direct methods
Least-squares matrix: full	Secondary atom site location: difference Fourier map
*R*[*F*^2^ > 2σ(*F*^2^)] = 0.035	Hydrogen site location: inferred from neighbouring sites
*wR*(*F*^2^) = 0.098	H-atom parameters constrained
*S* = 1.04	*w* = 1/[σ^2^(*F*_o_^2^) + (0.0518*P*)^2^ + 1.6228*P*] where *P* = (*F*_o_^2^ + 2*F*_c_^2^)/3
2787 reflections	(Δ/σ)_max_ = 0.001
175 parameters	Δρ_max_ = 0.42 e Å^−^^3^
0 restraints	Δρ_min_ = −0.25 e Å^−^^3^

### Special details

**Table:**

Geometry. All e.s.d.'s (except the e.s.d. in the dihedral angle between two l.s. planes) are estimated using the full covariance matrix. The cell e.s.d.'s are taken into account individually in the estimation of e.s.d.'s in distances, angles and torsion angles; correlations between e.s.d.'s in cell parameters are only used when they are defined by crystal symmetry. An approximate (isotropic) treatment of cell e.s.d.'s is used for estimating e.s.d.'s involving l.s. planes.
Refinement. Refinement of *F*^2^ against ALL reflections. The weighted *R*-factor *wR* and goodness of fit *S* are based on *F*^2^, conventional *R*-factors *R* are based on *F*, with *F* set to zero for negative *F*^2^. The threshold expression of *F*^2^ > σ(*F*^2^) is used only for calculating *R*-factors(gt) *etc*. and is not relevant to the choice of reflections for refinement. *R*-factors based on *F*^2^ are statistically about twice as large as those based on *F*, and *R*- factors based on ALL data will be even larger.

### Fractional atomic coordinates and isotropic or equivalent isotropic displacement parameters (Å^2^)

**Table:**

	*x*	*y*	*z*	*U*_iso_*/*U*_eq_	
O1	0.92545 (3)	0.44122 (8)	0.38295 (8)	0.01527 (19)	
O2	0.91010 (5)	0.09784 (10)	0.30367 (10)	0.0322 (3)	
O3	0.94389 (4)	0.20695 (9)	0.49626 (9)	0.0237 (2)	
O4	0.80179 (4)	0.21560 (10)	0.15093 (10)	0.0272 (2)	
O5	0.85951 (3)	0.23192 (9)	−0.01412 (9)	0.0206 (2)	
O6	0.84027 (4)	0.69996 (9)	0.08929 (10)	0.0250 (2)	
O7	0.80331 (4)	0.50766 (9)	0.00853 (10)	0.0227 (2)	
C1	0.97293 (4)	0.35610 (11)	0.20451 (11)	0.0133 (2)	
H1	0.9723	0.3106	0.1117	0.016*	
C2	0.92422 (4)	0.33021 (12)	0.28941 (11)	0.0141 (2)	
C3	0.87579 (4)	0.36255 (12)	0.18620 (11)	0.0149 (2)	
C4	0.87513 (5)	0.49467 (12)	0.17852 (12)	0.0157 (2)	
C5	0.92285 (4)	0.54296 (12)	0.27571 (11)	0.0149 (2)	
H5A	0.9202	0.6361	0.3103	0.018*	
C6	0.97323 (4)	0.51140 (11)	0.20141 (11)	0.0134 (2)	
H6	0.9748	0.5528	0.1071	0.016*	
C7	0.92448 (5)	0.19809 (12)	0.36304 (12)	0.0171 (3)	
C8	0.93932 (6)	0.08885 (15)	0.58051 (15)	0.0322 (3)	
H8A	0.9590	0.1018	0.6726	0.048*	
H8B	0.9543	0.0129	0.5338	0.048*	
H8C	0.9016	0.0721	0.5920	0.048*	
C9	0.84091 (5)	0.26211 (12)	0.10758 (12)	0.0165 (2)	
C10	0.82628 (5)	0.14106 (15)	−0.10214 (14)	0.0263 (3)	
H10A	0.8400	0.1345	−0.1942	0.039*	
H10B	0.7895	0.1742	−0.1134	0.039*	
H10C	0.8270	0.0533	−0.0582	0.039*	
C11	0.83860 (4)	0.58061 (13)	0.08826 (12)	0.0170 (2)	
C12	0.76407 (5)	0.58064 (14)	−0.08202 (15)	0.0266 (3)	
H12A	0.7429	0.5185	−0.1433	0.040*	
H12B	0.7824	0.6433	−0.1392	0.040*	
H12C	0.7404	0.6294	−0.0247	0.040*	

### Atomic displacement parameters (Å^2^)

**Table:**

	*U*^11^	*U*^22^	*U*^33^	*U*^12^	*U*^13^	*U*^23^
O1	0.0186 (4)	0.0149 (4)	0.0123 (4)	0.0003 (3)	0.0013 (3)	−0.0014 (3)
O2	0.0547 (7)	0.0168 (5)	0.0246 (5)	−0.0047 (4)	0.0011 (4)	−0.0007 (4)
O3	0.0295 (5)	0.0222 (5)	0.0183 (4)	−0.0033 (4)	−0.0035 (4)	0.0069 (4)
O4	0.0228 (5)	0.0311 (6)	0.0287 (5)	−0.0110 (4)	0.0078 (4)	−0.0079 (4)
O5	0.0188 (4)	0.0252 (5)	0.0177 (4)	−0.0039 (3)	0.0015 (3)	−0.0053 (4)
O6	0.0227 (5)	0.0180 (5)	0.0329 (5)	0.0026 (3)	−0.0045 (4)	−0.0004 (4)
O7	0.0180 (4)	0.0207 (5)	0.0274 (5)	−0.0002 (3)	−0.0091 (4)	0.0033 (4)
C1	0.0127 (5)	0.0146 (6)	0.0120 (5)	−0.0002 (4)	−0.0012 (4)	−0.0003 (4)
C2	0.0143 (5)	0.0150 (6)	0.0127 (5)	−0.0004 (4)	−0.0001 (4)	−0.0018 (4)
C3	0.0116 (5)	0.0190 (6)	0.0142 (5)	−0.0004 (4)	0.0009 (4)	0.0002 (4)
C4	0.0121 (5)	0.0188 (6)	0.0160 (5)	0.0002 (4)	0.0002 (4)	−0.0011 (4)
C5	0.0154 (5)	0.0138 (6)	0.0153 (5)	0.0013 (4)	−0.0004 (4)	−0.0001 (4)
C6	0.0133 (5)	0.0138 (6)	0.0126 (5)	−0.0004 (4)	−0.0019 (4)	−0.0001 (4)
C7	0.0166 (6)	0.0179 (6)	0.0174 (6)	0.0007 (4)	0.0038 (4)	0.0013 (5)
C8	0.0412 (8)	0.0286 (8)	0.0263 (7)	0.0004 (6)	0.0011 (6)	0.0140 (6)
C9	0.0139 (5)	0.0180 (6)	0.0172 (5)	0.0004 (4)	−0.0007 (4)	0.0001 (5)
C10	0.0243 (7)	0.0316 (8)	0.0223 (6)	−0.0046 (5)	−0.0013 (5)	−0.0102 (6)
C11	0.0125 (5)	0.0196 (6)	0.0188 (5)	0.0009 (4)	0.0013 (4)	0.0003 (5)
C12	0.0193 (6)	0.0274 (7)	0.0306 (7)	0.0005 (5)	−0.0106 (5)	0.0074 (6)

### Geometric parameters (Å, º)

**Table:**

O1—C2	1.4331 (14)	C3—C4	1.3343 (17)
O1—C5	1.4500 (14)	C3—C9	1.4960 (16)
O2—C7	1.1991 (16)	C4—C11	1.4808 (16)
O3—C7	1.3271 (15)	C4—C5	1.5275 (15)
O3—C8	1.4499 (16)	C5—C6	1.5435 (16)
O4—C9	1.1987 (15)	C5—H5A	1.0000
O5—C9	1.3331 (15)	C6—C6^i^	1.563 (2)
O5—C10	1.4547 (15)	C6—H6	1.0000
O6—C11	1.2043 (16)	C8—H8A	0.9800
O7—C11	1.3358 (15)	C8—H8B	0.9800
O7—C12	1.4514 (14)	C8—H8C	0.9800
C1—C1^i^	1.545 (2)	C10—H10A	0.9800
C1—C2	1.5553 (15)	C10—H10B	0.9800
C1—C6	1.5663 (16)	C10—H10C	0.9800
C1—H1	1.0000	C12—H12A	0.9800
C2—C7	1.5076 (16)	C12—H12B	0.9800
C2—C3	1.5295 (15)	C12—H12C	0.9800
			
C2—O1—C5	96.42 (8)	C5—C6—H6	115.8
C7—O3—C8	116.11 (11)	C6^i^—C6—H6	115.8
C9—O5—C10	114.99 (10)	C1—C6—H6	115.8
C11—O7—C12	116.12 (10)	O2—C7—O3	125.51 (12)
C1^i^—C1—C2	113.51 (11)	O2—C7—C2	122.33 (11)
C1^i^—C1—C6	90.30 (5)	O3—C7—C2	112.12 (10)
C2—C1—C6	100.56 (9)	O3—C8—H8A	109.5
C1^i^—C1—H1	116.2	O3—C8—H8B	109.5
C2—C1—H1	116.2	H8A—C8—H8B	109.5
C6—C1—H1	116.2	O3—C8—H8C	109.5
O1—C2—C7	113.46 (9)	H8A—C8—H8C	109.5
O1—C2—C3	101.97 (9)	H8B—C8—H8C	109.5
C7—C2—C3	117.36 (10)	O4—C9—O5	125.55 (11)
O1—C2—C1	103.01 (9)	O4—C9—C3	123.42 (11)
C7—C2—C1	115.14 (9)	O5—C9—C3	111.03 (10)
C3—C2—C1	104.05 (9)	O5—C10—H10A	109.5
C4—C3—C9	130.13 (11)	O5—C10—H10B	109.5
C4—C3—C2	104.70 (10)	H10A—C10—H10B	109.5
C9—C3—C2	125.05 (10)	O5—C10—H10C	109.5
C3—C4—C11	128.35 (11)	H10A—C10—H10C	109.5
C3—C4—C5	106.24 (10)	H10B—C10—H10C	109.5
C11—C4—C5	125.29 (11)	O6—C11—O7	125.15 (11)
O1—C5—C4	100.80 (9)	O6—C11—C4	124.12 (11)
O1—C5—C6	101.41 (9)	O7—C11—C4	110.72 (11)
C4—C5—C6	106.46 (9)	O7—C12—H12A	109.5
O1—C5—H5A	115.4	O7—C12—H12B	109.5
C4—C5—H5A	115.4	H12A—C12—H12B	109.5
C6—C5—H5A	115.4	O7—C12—H12C	109.5
C5—C6—C6^i^	114.97 (11)	H12A—C12—H12C	109.5
C5—C6—C1	101.06 (9)	H12B—C12—H12C	109.5
C6^i^—C6—C1	89.66 (5)		
			
C5—O1—C2—C7	−178.09 (9)	O1—C5—C6—C1	37.92 (10)
C5—O1—C2—C3	−50.92 (9)	C4—C5—C6—C1	−67.09 (10)
C5—O1—C2—C1	56.76 (9)	C1^i^—C1—C6—C5	−117.62 (10)
C1^i^—C1—C2—O1	62.75 (8)	C2—C1—C6—C5	−3.62 (10)
C6—C1—C2—O1	−32.23 (10)	C1^i^—C1—C6—C6^i^	−2.22 (12)
C1^i^—C1—C2—C7	−61.30 (10)	C2—C1—C6—C6^i^	111.78 (10)
C6—C1—C2—C7	−156.28 (9)	C8—O3—C7—O2	−10.06 (19)
C1^i^—C1—C2—C3	168.84 (6)	C8—O3—C7—C2	172.22 (11)
C6—C1—C2—C3	73.86 (10)	O1—C2—C7—O2	160.49 (12)
O1—C2—C3—C4	32.45 (11)	C3—C2—C7—O2	41.86 (17)
C7—C2—C3—C4	157.06 (10)	C1—C2—C7—O2	−81.16 (15)
C1—C2—C3—C4	−74.43 (11)	O1—C2—C7—O3	−21.71 (14)
O1—C2—C3—C9	−151.28 (10)	C3—C2—C7—O3	−140.34 (10)
C7—C2—C3—C9	−26.67 (16)	C1—C2—C7—O3	96.65 (12)
C1—C2—C3—C9	101.85 (12)	C10—O5—C9—O4	4.24 (18)
C9—C3—C4—C11	0.3 (2)	C10—O5—C9—C3	−176.27 (10)
C2—C3—C4—C11	176.35 (11)	C4—C3—C9—O4	−94.36 (17)
C9—C3—C4—C5	−175.80 (11)	C2—C3—C9—O4	90.36 (16)
C2—C3—C4—C5	0.21 (12)	C4—C3—C9—O5	86.14 (16)
C2—O1—C5—C4	50.47 (9)	C2—C3—C9—O5	−89.15 (13)
C2—O1—C5—C6	−58.97 (9)	C12—O7—C11—O6	−0.57 (18)
C3—C4—C5—O1	−32.25 (12)	C12—O7—C11—C4	178.21 (10)
C11—C4—C5—O1	151.47 (11)	C3—C4—C11—O6	178.87 (13)
C3—C4—C5—C6	73.20 (12)	C5—C4—C11—O6	−5.68 (19)
C11—C4—C5—C6	−103.08 (12)	C3—C4—C11—O7	0.08 (18)
O1—C5—C6—C6^i^	−56.92 (8)	C5—C4—C11—O7	175.53 (11)
C4—C5—C6—C6^i^	−161.93 (7)		

Symmetry code: (i) −*x*+2, *y*, −*z*+1/2.

### Hydrogen-bond geometry (Å, º)

**Table:**

*D*—H···*A*	*D*—H	H···*A*	*D*···*A*	*D*—H···*A*
C6—H6···O1^ii^	1.00	2.38	3.2105 (13)	140

Symmetry code: (ii) *x*, −*y*+1, *z*−1/2.
